# Host Membranes as Drivers of Virus Evolution

**DOI:** 10.3390/v15091854

**Published:** 2023-08-31

**Authors:** Mélanie Matveeva, Marine Lefebvre, Henri Chahinian, Nouara Yahi, Jacques Fantini

**Affiliations:** Department of Biology, Faculty of Medicine, University of Aix-Marseille, INSERM UMR_S 1072, 13015 Marseille, France; melanie.matveeva@etu.univ-amu.fr (M.M.); marine.lefebvre.1@etu.univ-amu.fr (M.L.); henrichahinian@gmail.com (H.C.); nouara.yahi@univ-amu.fr (N.Y.)

**Keywords:** lipid rafts, gangliosides, cholesterol, SARS-CoV-2, HIV-1, Monkeypox virus, electrostatic potential, hydrogen bond, evolution, mutations

## Abstract

The molecular mechanisms controlling the adaptation of viruses to host cells are generally poorly documented. An essential issue to resolve is whether host membranes, and especially lipid rafts, which are usually considered passive gateways for many enveloped viruses, also encode informational guidelines that could determine virus evolution. Due to their enrichment in gangliosides which confer an electronegative surface potential, lipid rafts impose a first control level favoring the selection of viruses with enhanced cationic areas, as illustrated by SARS-CoV-2 variants. Ganglioside clusters attract viral particles in a dynamic electrostatic funnel, the more cationic viruses of a viral population winning the race. However, electrostatic forces account for only a small part of the energy of raft-virus interaction, which depends mainly on the ability of viruses to form a network of hydrogen bonds with raft gangliosides. This fine tuning of virus-ganglioside interactions, which is essential to stabilize the virus on the host membrane, generates a second level of selection pressure driven by a typical induced-fit mechanism. Gangliosides play an active role in this process, wrapping around the virus spikes through a dynamic quicksand-like mechanism. Viruses are thus in an endless race for access to lipid rafts, and they are bound to evolve perpetually, combining speed (electrostatic potential) and precision (fine tuning of amino acids) under the selective pressure of the immune system. Deciphering the host membrane guidelines controlling virus evolution mechanisms may open new avenues for the design of innovative antivirals.

## 1. Introduction

To introduce their genetic material inside the target cells, enveloped viruses first cross the barrier constituted by the plasma membrane [1]. Two strategies are then possible: (i) the fusion of the viral envelope with the plasma membrane [2], or (ii) a process of endocytosis of the intact viral particle, which will later fuse with the endosome according to a pH-dependent mechanism [3]. These two mechanisms, if perfectly controlled, cannot be initiated by the random fixation of the virus on the membrane of the host. In fact, the viruses are attracted to privileged zones of fusion or endocytosis, referred to as lipid rafts [4,5], which concentrate the membrane components required for virus fusion or endocytosis, i.e., sphingolipids, cholesterol, and caveolin [6,7,8,9]. 

**Figure 1:**
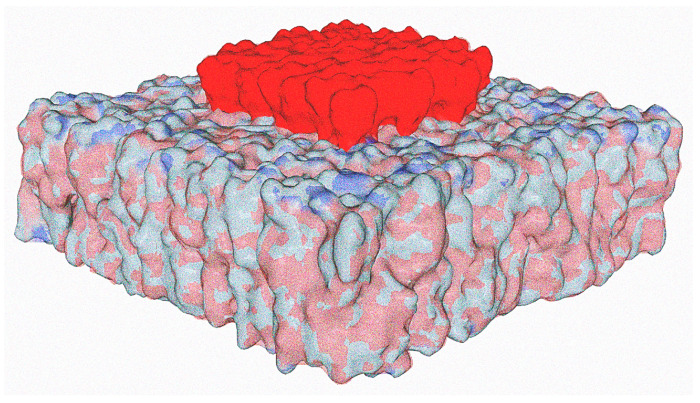
Biochemical organization and surface potential of a lipid raft. This schematic representation of a typical ganglioside/cholesterol raft was created for the present article based on molecular dynamics simulations of a ganglioside/cholesterol/phosphatidylcholine system [24]. We used the surface tool of Molegro Molecular Viewer to generate the electrostatic surface potential of the membrane as previously described [25]. A film grain was then applied with Microsoft Power Point picture tool artistic effects. Electronegative zones are in red, electropositive zones are in blue, and neutral zones are in white/grey. Note that the raft protrudes at the membrane surface, with a strong electronegative field due to anionic gangliosides. This difference in height is due to two structural characteristics that differentiate raft lipids (sphingolipids, including gangliosides) from bulk phase lipids (glycerophospholipids, including phosphatidylcholine). First, the kinked structure of the fatty acyl chains of phosphatidylcholine results in a shorter molecular length relative to the straight sphingolipid molecules. Accordingly, atomic force microscopy (AFM) can reveal sphingolipid rafts protruding from a phosphatidylcholine background [26]. Secondly, the large sugar moiety of gangliosides also contributes to the specific height of lipid raft domains [27].

From a biochemical point of view, lipid rafts are microdomains consisting of an assembly of cholesterol and sphingolipids in the outer leaflet of the plasma membrane (Figure 1). One of the most intriguing aspects of lipid rafts is that they generate an electronegative electrostatic field due to the presence of glycosphingolipids (rich in oxygen atoms) and gangliosides (anionic at physiological pH due to their sialic acids) [10]. The surface electrostatic potential is thus a critical parameter controlling virus-cell interactions [11,12,13,14,15,16,17,18]. The other key property of lipid rafts is the enrichment in cholesterol, a critical cofactor for virus fusion and endocytosis [19]. The requirement of cholesterol in virus entry has been demonstrated by the inhibition of infection in cells treated by the cholesterol-depleting agent methyl-β-cyclodextrin or statins [20,21,22,23].

Lipid rafts are dynamic membrane entities that can fuse and modify their shape just like “a myriad of mercury sheets perpetually moving on the surface of a mirror” [28]. Indeed, the term “lipid rafts” generally refers to “a collection of domains that differ in protein and lipid composition as well as in temporal stability” [29]. In 2006, the Keystone Symposium on Lipid Rafts and Cell Function brought together experts in biophysics, biochemistry, and cell biology to discuss the structure and function of lipid rafts [30]. A consensus emerged to define the rafts as small (10–200 nm), heterogeneous, and highly dynamic membrane microdomains enriched in sterol and sphingolipids. As this review is essentially devoted to the role of lipid rafts in the adhesion of respiratory viruses to the plasma membrane of host cells, two important questions must first be addressed: (i) is the average size of the rafts compatible with the size of the viral spikes, and (ii) do the cells of the respiratory epithelium have an appropriate raft density to ensure virus adhesion?

**Figure 2:**
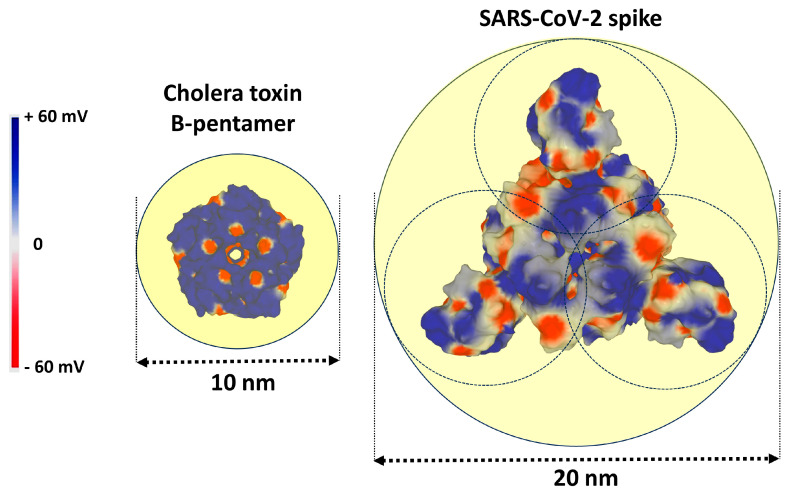
Relationship between the size of the lipid rafts and the size of a raft targeting microbial structures. A raft of 10 nm in diameter can easily accommodate a cholera toxin B-pentamer. A raft of 20 nm in diameter (or three rafts of 10 nm) can accommodate a trimeric spike of SARS-CoV-2. In both cases, the raft area is represented by a yellow disk. The electrostatic surface potential of the proteins is colored in blue (electropositive), red (electronegative), and white (neutral). The electrostatic potential scale is in mV (from −60 to +60). The distances were determined with Swiss PDB Viewer [39]. The structure of the cholera toxin B-pentamer was retrieved from pdb 3CHB [31]. The structure of SARS-CoV-2 trimer was retrieved from pdb 6VSB [40]. Both structural models were generated with the surface tool Molegro Molecular Viewer [25].

The problem of the size of the rafts in relation to the size of the spikes of SARS-CoV-2 is illustrated in Figure 2. Assuming that the minimum size of a raft is 10 nm in diameter [30], we see that the cholera toxin B-pentamer is perfectly compatible with this raft, since its diameter is about 6 nm when bound to ganglioside GM1 [31,32]. The diameter of the trimeric spike of SARS-CoV-2 is approximately 15 nm [33]. Therefore, the viral spike requires a larger raft, such as the 20 nm diameter one shown in Figure 2. Alternatively, we can assume that the trimeric spike can successively interact with three distinct 10 nm diameter rafts that could be recruited during a cooperative adhesion process. In all cases, it appears that the current size of the rafts (10–100 nm) is well adapted to the spike of SARS-CoV-2.

Lipid rafts of respiratory epithelial cells play a critical role in respiratory infections. The alveolar epithelium is the largest epithelial surface of the host exposed to the external environment. Its surface has been estimated to be 140 m^2^ which can be compared to the size of a tennis court [34,35]. The principal cell type (up to 95%) of the alveolar epithelium is type I pneumocyte [36], which performs gas exchange in the lungs. Correspondingly, type I pneumocytes are constantly exposed to the external environment and pathogens. The cell membrane of type I pneumocytes has a high density of lipid rafts that occupy more than two thirds of the plasma membrane [34]. Such a high concentration of rafts is perfectly suited to attract many pathogens, including respiratory viruses such as the influenza virus [37] and SARS-CoV-2 [38].

## 2. Lipid Rafts and SARS-CoV-2 Entry: Key Role of Surface Potential

Most—if not all—enveloped viruses use lipid rafts as privileged attachment sites on the plasma membrane of host cells [41,42]. This is particularly the case for coronaviruses such as SARS-CoV-2 [43,44]. There are many advantages to selecting rafts to fracture the membrane barrier that protects the cell from microbiological aggressors. First, the surface potential of the rafts helps to concentrate the viral particles in delimited zones of the membrane, which favors the reduction of dimensionality of the 3D volume towards the 2D space represented by the cell surface [45]. This may give viruses a kinetic advantage over other potential extracellular ligands, including competing viruses whose surface potential is too weakly electropositive [25]. Secondly, as discussed above, the rafts are enriched in cholesterol, an essential co-factor for the mechanism of virus-cell fusion [46]. Thus, the ganglioside-cholesterol pair, which constitutes the basic unit of lipid rafts, plays a dual role in viral infection: attachment (ganglioside) and fusion (cholesterol) [47]. The 1:1 stoichiometry (mol:mol) of these two partners is perfectly suited to this dual function of rafts. It is interesting to note that the electronegative surface potential of rafts is chiefly due to gangliosides (and not cholesterol) in perfect harmony with the role of gangliosides in the attraction and initial attachment of viral particles to the surface of the target cells [48]. But even at this initial stage in which cholesterol does not play a direct role, this lipid intervenes indirectly by controlling the conformation of the sugar part of the gangliosides. Thus, it is indeed the ganglioside-cholesterol couple which is recognized in an optimal manner by the glycoproteins of the virus envelope. In this mechanism, cholesterol exerts a kinetic effect by accelerating the binding of viruses to raft gangliosides. This process applies to viruses, but also to amyloid proteins which interact with the plasma membrane of brain cells at the level of lipid rafts, while also taking advantage of the particular properties of the ganglioside-cholesterol couple [49].

**Figure 3:**
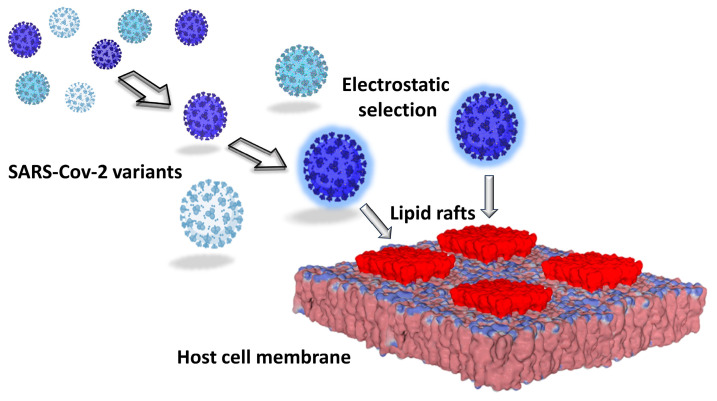
How electrostatic forces may control the kinetics of virus infection and select the viruses with a strong surface potential.

Seen from the outside, the plasma membrane of the host cell is studded with slightly protruding plates that display a high electronegative potential (Figure 1). At first glance, this static representation (the rafts are here considered inert) makes it possible to understand why the most electropositive viruses win the race to the cell surface (Figure 3). Indeed, a large body of experimental data has shown that electrostatic attraction between viruses and oppositely charged absorbent surfaces results in rapid and extensive adsorption [50,51,52,53]. Conversely, electrostatic repulsion between viruses and similarly charged absorbent surfaces delays or completely inhibits adsorption [52,54,55,56]. A particularly interesting study has made it possible to compare the electrostatic attraction of viruses with similar sizes and shapes but differing in their surface charges and polarities. In this case, the authors were able to demonstrate the critical importance of electrostatic interactions in virus-sorbent attraction [52]. In this context, lipid rafts can be assimilated to electrostatic landing strips for viruses [48], which is perfectly valid, but, as we will see later, far too schematic and incomplete. Nevertheless, the surface potential does exert a selection pressure on viruses in connection with the physicochemical conditions of the rafts.

The electronegative charges of lipid rafts are due to the sialic acid units of gangliosides which bear an anionic carboxylate at physiological pH. The binding to sialic acids has been demonstrated for several coronaviruses [57,58,59,60,61], MERS-CoV [62,63] and SARS-CoV-2 [64,65,66,67]. Incidentally, other negatively charged surface components such as heparan sulfate can serve as virus attachment sites [68,69,70,71,72]. Indeed, sulfated polymers provide an electronegative field functionally comparable to lipid rafts [18], although not as conformationally flexible due to polymeric structure constraints [73], such as a limited range of glycosidic linkage geometries in the repeating disaccharides [74]. The propensity of viruses to interact with negatively charged structures on the surface of host cells explains the antiviral effects of anionic compounds such as heparin [75], pentosan sulfate [76], suramin [77], or sulfated glycomimetics [78].

**Figure 4:**
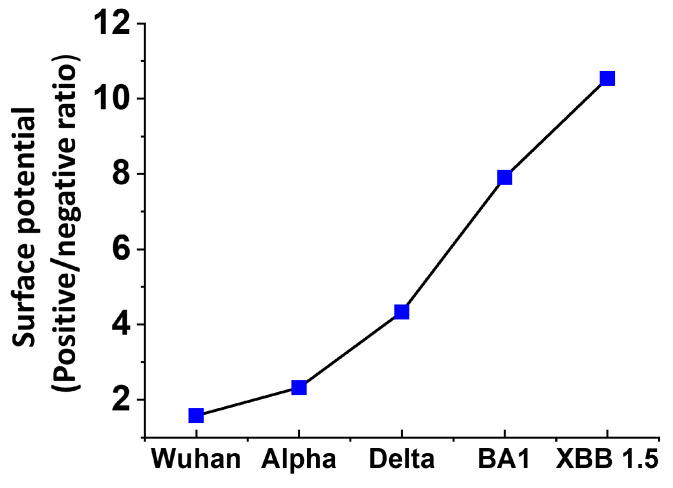
Electrostatic surface potential of representative SARS-CoV-2 variants (calculated on the spike trimer surface facing the host cells by integration of the positive (blue) and negative (red) areas and expressed as the positive/negative ratio (values from ref. [25]), completed with the value obtained for Omicron XBB 1.5 calculated with the same method for the present article).

The analysis of the surface potential of the successive variants of SARS-CoV-2 perfectly illustrates the peremptory nature of this selection pressure. As shown in Figure 4, there is a gradual increase in the electrostatic potential of the spike protein trimers [25] which, after three years of evolution, has been multiplied by 6.7 between the initial strain from Wuhan and the latest circulating variants in the summer of 2023, Omicron XBB 1.5 (Table 1). This evolution of the surface electrostatic potential is not only due to the accumulation of mutations in both the NTD and the RBD [25,79,80,81] but also to deletions in the NTD [25,82,83]. Among the mutations that increase the surface potential, one can cite E484K [84], which induces the simultaneous loss of a negative charge with a gain of a cationic charge [85]. Deletions in the NTD condense the electropositive areas, which may allow a faster access to lipid rafts [25].

## 3. Mutated Spike Proteins Combine Speed and Precision

Yet it would be too simple to believe that virus evolution would only consist of a gradual enrichment of cationic amino acids. For instance, the N-terminal domain (NTD) of the Delta variants was already highly electropositive [25]; therefore, further increases were highly improbable [11]. Indeed, the “super-Delta” virus never happened, and its decline allowed the emergence of a new lineage, Omicron [86]. This evolution by jumping from one lineage of variants to another clearly shows that the surface electrostatic potential is not the only parameter governing the evolution of viruses. As critical as it is, this parameter only accounts for the kinetics of virus-cell interactions, not for their quality, which depends on the avidity of the spike protein trimers for the rafts, but also on their affinity for the ACE2 receptor [25]. We must consider all the parameters (kinetics, avidity, and affinity) for estimating a transmissibility index (T-index) capable of predicting the supremacy of a line of SARS-CoV-2 variants on another [25]. The T-index has worked remarkably well in anticipating and predicting the penetration rate of SARS-CoV-2 variants in the Wuhan, Alpha, and up to the Delta series. The appearance of Omicron was a game-changer, because this virus can infect cells by two distinct mechanisms, classic membrane fusion (well characterized by the T-index) and endocytosis (not considered by the T-index). These discrepancies have been the subject of a complete study [86].

If the surface electrostatic potential of the spike trimers was sufficient to explain the evolution of SARS-CoV-2 and the emergence of new variants, one could expect that spike-ganglioside interactions mainly involve cationic amino acids, i.e., arginine and lysine. In fact, analysis of amino acid residues directly involved in virus-raft interactions shows that cationic residues contribute less than 15% to the overall energy of the interaction (Table 1).

This analysis shows that the ganglioside-spike interaction chiefly involves amino acids able to form hydrogen bonds [88,89], such as serine, tyrosine, asparagine, and glutamine. Moreover, anionic residues (aspartate and glutamate), which can also form hydrogen bonds [90] with the sugar part of gangliosides, significantly contribute to the binding [91].

**Figure 5:**
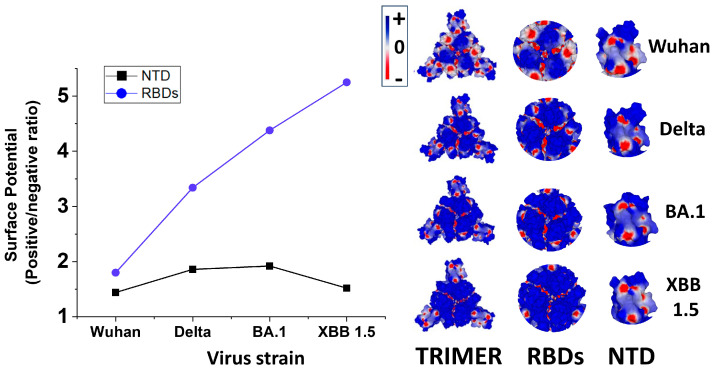
Comparative evolution of the electrostatic surface potential of SARS-CoV-2 variants in the central zone of the spike trimer (RBDs) and on lateral NTDs (retrieved from ref. [92] and completed with the value of Omicron XBB 1.5 calculated with the same method). Electropositive and electronegative areas are colored in blue and red, respectively. Neutral areas are in white. The electrostatic potential, calculated as the sum of the Coulomb potentials for each atom of the considered molecule, with a distance-dependent dielectric constant l, was visualized by Molegro Molecular Viewer [11]. The electrostatic potential scale is in mV (from −60 to +60).

According to these data, the electrostatic surface potential of the spike protein appears to be totally independent of its hydrogen bond forming capacity. The evolution of the surface potential of trimeric spikes has been compared among SARS-CoV-2 variants from the original Wuhan strain to Omicron BA.1 [92]. It appeared that the surface potential changes do not concern the whole surface but preferentially the central areas which correspond to the masked receptor binding domains (RBD) in the prefusion conformation. The data of Figure 4 and Figure 5 perfectly reflect this phenomenon, since the gradual increase of the surface potential is chiefly due to the RBD. In contrast, the N-terminal domain (NTD) has a distinctive mode of evolution: regular increase of the surface potential from Wuhan to Delta [25,93], then stabilization between Delta and Omicron BA.1 [86], and finally, a decrease between BA.1 and XBB 1.5, the dominant variant in the summer of 2023 [94] (Figure 5). As discussed above, this discrepancy emphasizes a dual property of Omicron: stabilization of the surface potential in the NTD, but strong increase in the RBD so that globally, the trimeric spike of Omicron is significantly more positive than Delta [92].

This can be viewed as a compensation mechanism in which above a critical level of electropositivity, the NTD transfers the electrostatic guideline given by the host membrane to the RBD. The advantage of this transfer is double: (i) the NTD does not stick too strongly to the raft by nonproductive electrostatic interactions and thus retains the conformational flexibility required for the formation of the hydrogen bond network between the trimeric spike and the raft; (ii) the increased electropositivity of the RBD is fully compatible with the highly electronegative surface of the ACE2 receptor [25], a property that has recently been recognized as a key parameter determining the infection of humans and animals by SARS-CoV-2 variants [95,96,97]. The association of ACE2 in lipid rafts [23,98] is an important parameter that facilitates the recruitment of this receptor soon after the initial interaction of the NTD with the host cell surface. The journey of the bound virus from a raft without ACE2 to a raft with ACE2 can be mediated by a lateral diffusion that has been compared to surfing [99]. It can also be interpreted as a two-receptor mechanism [100] ensuring the raft-to-raft transfer of CD4-bound HIV-1 to CCR5 coreceptors [48]. In this respect, the dynamics of lipid rafts on the cell surface are a critical parameter that controls the transition from the adhesion step to the fusion machinery [41,101,102,103,104]. Similarly, the lifetime of rafts [105,106,107] and its connection with the kinetics of virus binding [108] warrants future studies using non-destructive approaches [109]. Interestingly, it has been shown that the density of lipid raft on the surface of respiratory cells can be modulated by extracellular vesicles in COVID-19 patients, promoting viral entry [110]. These observations further emphasize the cross-talk between the virus and the plasma membrane.

**Figure 6:**
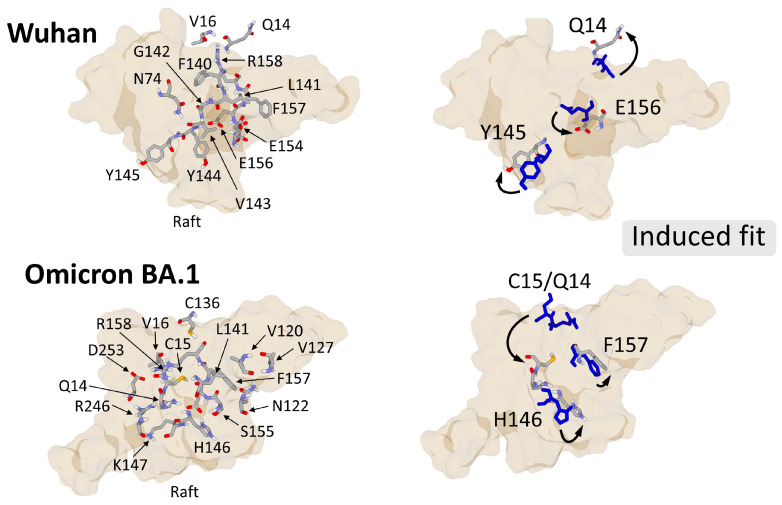
Fine tuning of virus-raft interactions illustrated by the Wuhan and Omicron BA.1 strains of SARS-CoV-2. In the left panels, all NTD amino acid residues forming a network of hydrogen bond with raft gangliosides are represented above the raft. In the right panel, one can see a selection of typical conformational changes (arrows) occurring in the NTD before (blue sticks) or after (atomic colored sticks) binding to raft gangliosides. The conformational changes allowing the lipid raft to accommodate both the Wuhan and Omicron NTD (quicksand effect) are clearly visible by comparing the raft shapes in the upper (Wuhan) and lower (Omicron BA.1) panels. These data were retrieved from refs. [25,86].

An illustration of hydrogen bonds in the NTD-ganglioside complex of the Wuhan and XBB 1.5 strains is presented in Figure 6. The conformational flexibility and adaptability of the same ganglioside raft to the NTD of the Wuhan and Omicron BA.1 strains of SARS-CoV-2 is somewhat spectacular. After being attracted by the electronegative field at the speed control corresponding to the electropositive level of the spike trimers, the NTD slightly penetrates the raft, which wraps around it by engaging several high energy hydrogen bonds. We can thus compare the raft to a transient mold whose shape is able to adapt to more rigid structures such as a trimeric spike. The shape and distribution of electrical charges is different for each variant trimer (Figure 5), yet the particular structure of the raft, made of a multitude of ganglioside/cholesterol units, has enough flexibility to adapt to each type of trimeric spike. Due to its greater rigidity (compared to the higher flexibility of raft gangliosides), the trimer will then sink slightly into the raft, as if it was interacting with quicksand. For this reason, we propose to name this reorganization of the raft the quicksand effect. The coordinated movements of the amino acid side chains of the NTD and of the sugars of ganglioside headgroups are a typical example of fine conformational tuning though a sequentially controlled induced fit mechanism. These interfacial adjustments of the raft and of the NTD are facilitated by surrounding water molecules controlling the hydration-dehydration balance.

Overall, mutated spike proteins combine speed and precision to interact optimally with lipid rafts and trigger the fusion (or endocytosis) process leading to the penetration of the virus RNA in the host cell. Speed and precision are governed by lipid rafts and can be considered as mandatory membrane guidelines of virus evolution.

## 4. Membrane Guidelines of Virus Evolution

The induced fit mechanism that controls the functional interaction of the NTD of a given variant with raft gangliosides can be visualized by molecular modeling approaches (Figure 6). As discussed above, the conformational changes that optimize the binding of the NTD to the raft concerns both the gangliosides and key amino acid residues of the NTD [111]. For example, we can highlight a 180° reversal of the aromatic ring of tyrosine Y145 (Figure 6, upper right panel). In the case of Omicron BA.1, the conformational rearrangement implies a greater movement of the peptide chain, which can be visualized by the displacement of residues Q14 and C15 (Figure 6, lower right panel). Other adjustments are obtained by a reorientation of the side chain of residues such as Q14 and E156 (Wuhan NTD) or H146 and F157 (Omicron BA.1 NTD).

**Figure 7:**
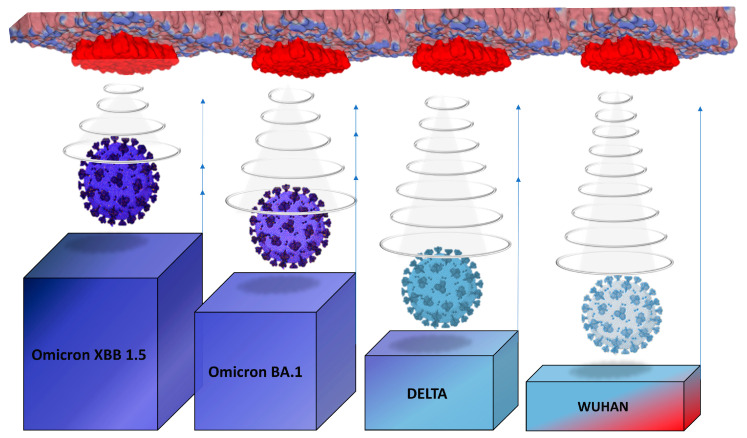
The selection of viruses by the electrostatic surface potential can be interpreted as an advantage given to the most electropositive viruses which win the race for access to rafts. The winner is the one with the most electropositive surface potential, which may account for the evolution of SARS-CoV-2 from the original Wuhan strain to the last variant circulating in the summer of 2023 (Omicron XBB 1.5).

**Figure 8:**
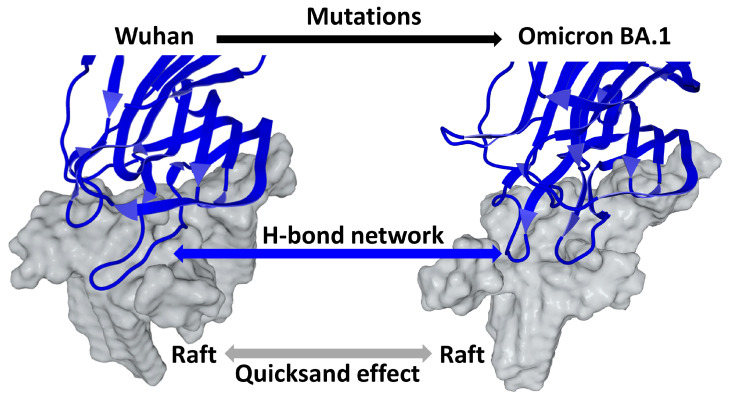
Fine-tuning virus selection operates on the fastest viruses previously selected by their surface electrostatic potential. This mechanism applies to the amino acid residues of the NTD, which can accumulate mutations as long as these remain compatible with the establishment of a stabilizing network of hydrogen bonds. However, this process requires a conformational reorganization of both the peptide chain and amino acid residues. The lipid raft plays an active role in these conformational adjustments. Its malleable surface can be compared to quicksand absorbing the surface of the more rigid NTD, optimizing its attachment to the host cell membrane. All these phenomena can then be described as an induced fit mechanism involving the spike protein and the raft. Overall, these interfacial phenomena are facilitated by the hydration-dehydration balance of the surface of the raft and of the NTD. The structural models of raft-NTD interactions are those published in [25,86] for the Wuhan and Omicron strains, respectively. The raft surface is colored in gray and the NTD backbone in blue. The models have been created with Molegro Molecular Viewer [25].

Overall, it can be hypothesized that the host membrane exerts a selective pressure governing both the kinetics (Figure 7) and precision (Figure 8) of the virus-raft binding process. This pressure of selection can be translated in a list of guidelines for mutant viruses under penalty of being eliminated.

Mutations that are not compatible with this two-step mechanism are likely forbidden because they will lead to variants that are either too slow (if the electrostatic potential is decreased) or unable to lock the complex (if not compatible with the stabilizing hydrogen bond network). For instance, the substitution of anionic (Asp or Glu) by cationic (Arg or Lys) residues is generally favored because it will render the virus more electropositive. A similar effect is obtained when an anionic residue is replaced by a neutral one, or when a glycosylation site is eliminated, as observed for the V3 loop of the HIV-1 surface envelope glycoprotein [48].

However, these possibilities remain limited, at least for the domains of attachment to rafts. Indeed, the positive charges of the Lys or Arg residues are not the most suitable for forming hydrogen bonds with ganglioside sugars because they can also be involved in electrostatic and/or cation-Pi interactions with proximal amino acid residues of the protein [112]. Their long side chain that ends with the cationic group favors the folding of these residues toward the protein surface, making them unavailable to interact with ganglioside headgroups. This may explain why the surface electrostatic potential of the NTD, after having reached its maximum level with the Delta variants, no longer increased, and even decreased in the Omicron series. At the same time, the network of hydrogen bonds that stabilizes the interaction of the virus with the raft was maintained, despite the accumulation of mutations (Figure 6).

The establishment of this network of hydrogen bonds requires an adaptation of the raft which appears to play an active role in the formation of the virus-cell complex. It is therefore essential that the raft is not a rigid structure but on the contrary, a malleable entity capable of offering the hydrogen bond-forming residues of the NTD many possibilities to reach the donor or acceptor atoms and form high energy hydrogen bonds. This conformational plasticity of lipid rafts has been shown by molecular modeling in the case of amyloid proteins [49]. Our team also highlighted it for viruses, as explained in Figure 8. This illustration brings together three distinct phenomena that describe the impact of fine-tuning in the selection of viruses: (i) the appearance of mutations that generate variants; (ii) the compatibility of these mutations with the formation of a hydrogen bonding network stabilizing the virus-cell complex, and (iii) the quicksand effect which accounts for the exceptional conformational plasticity of the raft which can accommodate highly mutated viruses.

The problematic of reconciling two selection pressures which do not always go in the same direction is perplexing. Only a high mutational potential can solve such an equation, and this is probably what happened with SARS-CoV-2 (and more generally for RNA viruses). Since its emergence at the end of 2019, SARS-CoV-2 has evolved into a myriad of variants constituting several successive lineages. Yet, for all these variants, the interactions of the viral particle with the host membrane remain a speed/precision affair, mixing immediate electrostatic attraction (love at first sight) and fine tuning (formation of a lasting couple). In this scenario, the lipid raft plays the matchmaker who sets the rules of the game. In parallel, immune evasion together with a greater potential to adhere to primary nasal epithelial tissue probably allowed the emergence and success of the Omicron lineage [113], given the high mutation rate of RNA viruses [114].

Finally, the membrane guidelines also apply to animal reservoirs, and to the back and forth of the virus from man to animals and from animals to man [95,96,97,115]. Yet in this case, it is rather the polymorphism of the ACE2 receptor, which has been highlighted as the driving force of the mutations optimizing RBD-ACE2 binding [97]. Lipid rafts do not play a direct role in these adaptations, but rather an indirect role because the ACE2 protein is associated with the rafts [23]. However, we have only a few data on the composition and the physicochemical properties of the rafts of the animal reservoirs and of their gangliosides. Progress in this field is therefore necessary to better understand the membrane guidelines that apply to the transmission of these viruses from human to animal and backwards.

## 5. Conclusions and Perspectives

In this review, we reconsider the role of lipid rafts in virus infection and evolution. These membrane microdomains are not just passive gateways through which viruses penetrate the cells, but rather, they play a more active role than generally admitted. Their unique biochemical composition confers them a series of emergent properties driving virus infection and evolution. These emergent properties are generated by the coalescence and conformational adaptability of ganglioside-cholesterol complexes, which are the molecular unit of lipid rafts.

Correspondingly, there is a permanent race that favors viruses with an increased surface electrostatic potential together with the capability of forming a network of hydrogen bonds with raft gangliosides. The immune system of the host also contributes to the selection of such virus variants. Identifying membrane guidelines governing the evolution of viruses is not only of an academic interest but also has prophylactic and therapeutic implications. In the case of SARS-CoV-2, both the NTD and RBD display neutralizing epitopes, which are generally located on the trimeric spike surface that faces the host cell membrane. Indeed, there is a significant overlapping between these neutralizing epitopes and the ganglioside and ACE2 binding domain [81,93]. The rules that determine the interaction of SARS-CoV-2 variants with host membrane concern the surface of the trimeric spikes that faces the host membrane. These areas constitute the interface between the plasma membrane and the virus envelope. Thus, it is not surprising that it also contains the NTD and RBD epitopes recognized by neutralizing antibodies [93]. In SARS-CoV-2 variants, most mutations (including deletions) are located in these zones and affect the recognition and neutralizing efficiency of antibodies, especially those elicited by vaccine formulations based on the Wuhan spike protein [93].

Neutralizing antibodies directed against HIV-1 [48] and the Monkeypox virus [116] also overlap ganglioside-binding and/or receptor binding domains. The accumulation of mutations in these domains are known to decrease the efficiency of neutralizing antibodies, especially for RNA viruses such as HIV-1. Some antiviral molecules can also inhibit host-virus interactions by competitive inhibition of ganglioside binding. This explains the broad antiviral properties of compounds such as hydroxychloroquine, azithromycin, and anionic polymers [117,118,119].

Deciphering the host membrane guidelines controlling virus evolution mechanisms may open new avenues for optimizing vaccine formulations and designing new antivirals.

## Figures and Tables

**Table 1:** Comparison of virus-raft interactions in SARS-CoV-2 variants. ^1^ Calculated on the trimeric spike surface facing the host cell (electropositive/electronegative ratio). ^2^ Determined from NTD-raft interaction data. ^3^ Contribution of Arg and Lys residues to the NTD-raft interaction. ^4^ Contribution of Asp and Glu residues to the NTD-raft interaction. These data were retrieved from refs. [25,86,87], except for XBB 1.5 (obtained by superposition of the NTD-raft complex from ref. [86]).

Variant	Surface Potential ^1^	Total Raft ^2^ (kJ/mol)	Cationic ^3^ (kJ/mol)	Anionic ^4^ (kJ/mol)
**Wuhan**	1.58 (1.0)	−401 (100%)	−38.8 (9.7%)	−59.4 (14.8%)
**Delta**	4.33 (× 2.7)	−402 (100%)	−43.8 (10.9%)	−70.2 (17.5%)
**Omicron BA.1**	7.91 (× 5.0)	−430 (107%)	−53.6 (12.5%)	−53.1 (12.3%)
**Omicron** **XBB 1.5**	10.54 (× 6.7)	−445 (110%)	−62.5 (14.0%)	−68.5 (15.4%)

## Data Availability

Not applicable.
